# Supplementary material for: Genomic epidemiology analysis of drug-resistant Mycobacterium tuberculosis distributed in Mexico
Source: PLoS One. 2023 Oct 13;18(10):e0292965. doi: 10.1371/journal.pone.0292965 (PMC10575498; doi:10.1371/journal.pone.0292965)
Supplement: Attachment — Submitted filename: response to reviewers_Mejia Ponce et al_2.docx [file pone.0292965.s007.docx]

Monday, September 12^th^, 2023

PLOS ONE

Dear Editors at PLOS ONE,

Please find attached a resubmission of the manuscript entitled “**Genomic epidemiology analysis of drug-resistant *Mycobacterium tuberculosis* distributed in Mexico”** (PONE-D-23-08010).

We thank you and the reviewers for your patience. We are pleased that reviewers find value in our research, mostly in the need of reporting *Mtb* genomes from geographic locations that are not included in global catalogues. In the new version of the manuscript, we have amended the document format according to PLOS ONE guidelines and have attended the minor concerns from both reviewers.

Please find below a point-by-point review for all the reviewer´s comments.

Sincerely,

**Dr. Cuauhtémoc Licona Cassani**

School of Engineering and Sciences

Tecnológico de Monterrey

[clicona@tec.mx](mailto:clicona@tec.mx)

**Journal Requirements:**

1. Please ensure that your manuscript meets PLOS ONE's style requirements, including those for file naming. The PLOS ONE style templates can be found at

<https://journals.plos.org/plosone/s/file?id=wjVg/PLOSOne_formatting_sample_main_body.pdf>

and

<https://journals.plos.org/plosone/s/file?id=ba62/PLOSOne_formatting_sample_title_authors_affiliations.pdf>

We have reviewed PLOS ONE style requirements and have modified the manuscript accordingly.

2. Thank you for providing the ethics approval documents. It seems the approval numbers in the documents do not fully match the ones in your ethics statement (R-2005-3301-18, and R-2013-785-001). Please comment on this and clarify if there are any missing ethics documents.

In addition, please provide copies of their English translation and clarify if clinical samples of Mtb were collected by any of the authors.

Please also indicate if the provided ethics approval documents have an expiration date and clarify why data access (in Novermber 2022) is a few years after the ethics approval (in 2018).

Thanks for the observation. We modified the ethics statement (See below) and now all the missing ethics documents are provided (in English).

The data access was made possible until 2022 due to financial problems encountered in the laboratory, including laboratory restrictions due to COVID-19 pandemic.

## *“Ethical concerns*

*We obtained informed consent (written) from all participants and/or their legal guardian(s). All the information collected from TB patients was treated confidentially. Sampling was carried out without physical intervention, with full care given to the patient's integrity. None of the authors were involved in the sampling procedure. The study was approved by the National Scientific Research and Ethics Committee of the IMSS, Mexico (R-2005-3301-18, R-2013-785-001 and R-2018-785-118). The ruling for each ethics protocol is valid for one year upon specific request of an extension. All the methods were performed in accordance with the guidelines and regulations established by the IMSS ethics committee.”*

3. We note that the grant information you provided in the ‘Funding Information’ and ‘Financial Disclosure’ sections do not match. When you resubmit, please ensure that you provide the correct grant numbers for the awards you received for your study in the ‘Funding Information’ section.

Thanks for the observation. Changes have been made accordingly.

4. Thank you for stating the following financial disclosure:

"We are also grateful for the financial support provided by several grants, including the Mexican National Council for Science and Technology (CONACYT) - Ciencia de Frontera – 319590 to CLC; postgraduate scholarships to PMMP and AARG from the CONACYT. Additionally, we are thankful to Tecnologico de Monterrey for their Seed Grant to CLC, MDD and AS, which supported genome sequencing. We would also like to acknowledge StrainBiotech for their contribution of laboratory supplies, which greatly facilitated our research."

Please state what role the funders took in the study. If the funders had no role, please state: "The funders had no role in study design, data collection and analysis, decision to publish, or preparation of the manuscript." If this statement is not correct you must amend it as needed.

Please include this amended Role of Funder statement in your cover letter; we will change the online submission form on your behalf.

Thanks for the observation. We have modified the “financial information” in the submission platform.

In addition, we would like to mention that, as stated in the “author contribution” section, all funder authors participated actively in the work.

5. Thank you for stating the following in the Acknowledgments Section of your manuscript:

"..We are also grateful for the financial support provided by several grants, including the Mexican National Council for Science and Technology (CONACYT) - Ciencia de Frontera – 319590 to CLC; postgraduate scholarships to PMMP and AARG from the CONACYT. Additionally, we are thankful to Tecnologico de Monterrey for their Seed Grant to CLC, MDD and AS, which supported genome sequencing.."

We note that you have provided funding information that is not currently declared in your Funding Statement. However, funding information should not appear in the Acknowledgments section or other areas of your manuscript. We will only publish funding information present in the Funding Statement section of the online submission form.

Please remove any funding-related text from the manuscript and let us know how you would like to update your Funding Statement. Currently, your Funding Statement reads as follows:

"We are also grateful for the financial support provided by several grants, including the Mexican National Council for Science and Technology (CONACYT) - Ciencia de Frontera – 319590 to CLC; postgraduate scholarships to PMMP and AARG from the CONACYT. Additionally, we are thankful to Tecnologico de Monterrey for their Seed Grant to CLC, MDD and AS, which supported genome sequencing. We would also like to acknowledge StrainBiotech for their contribution of laboratory supplies, which greatly facilitated our research."

Please include your amended statements within your cover letter; we will change the online submission form on your behalf.

Thanks for the observation. We have modified the acknowledgment section as follow:

# ***“Acknowledgements***

*We would like to express our deepest gratitude to the individuals who generously participated in this study, as well as to the clinical personnel at all sample collection sites for their invaluable assistance. Additionally, we are thankful to the Centro de Biotecnologia FEMSA from Tecnologico de Monterrey and StrainBiotech to provide support to perform the research.”*

6. In your Data Availability statement, you have not specified where the minimal data set underlying the results described in your manuscript can be found. PLOS defines a study's minimal data set as the underlying data used to reach the conclusions drawn in the manuscript and any additional data required to replicate the reported study findings in their entirety. All PLOS journals require that the minimal data set be made fully available. For more information about our data policy, please see <http://journals.plos.org/plosone/s/data-availability>.

Upon re-submitting your revised manuscript, please upload your study’s minimal underlying data set as either Supporting Information files or to a stable, public repository and include the relevant URLs, DOIs, or accession numbers within your revised cover letter. For a list of acceptable repositories, please see <http://journals.plos.org/plosone/s/data-availability#loc-recommended-repositories>. Any potentially identifying patient information must be fully anonymized.

Important: If there are ethical or legal restrictions to sharing your data publicly, please explain these restrictions in detail. Please see our guidelines for more information on what we consider unacceptable restrictions to publicly sharing data: <http://journals.plos.org/plosone/s/data-availability#loc-unacceptable-data-access-restrictions>. Note that it is not acceptable for the authors to be the sole named individuals responsible for ensuring data access.

We will update your Data Availability statement to reflect the information you provide in your cover letter.

We have modified the submission, so all the data is available. We additionally modified the Data Availability statement in the manuscript as follow:

“The raw WGS data generated in this study can be accessed in the NCBI BioProject database under the accession number PRJNA824124 (https://www.ncbi.nlm.nih.gov/bioproject/?term=PRJNA824124).”

7. We note that you have stated that you will provide repository information for your data at acceptance. Should your manuscript be accepted for publication, we will hold it until you provide the relevant accession numbers or DOIs necessary to access your data. If you wish to make changes to your Data Availability statement, please describe these changes in your cover letter and we will update your Data Availability statement to reflect the information you provide.

We have modified the submission, so all the data are available. We additionally modified the Data Availability statement in the manuscript as follow:

“The raw WGS data generated in this study can be accessed in the NCBI BioProject database under the accession number PRJNA824124 (https://www.ncbi.nlm.nih.gov/bioproject/?term=PRJNA824124).”

8. Please amend either the abstract on the online submission form (via Edit Submission) or the abstract in the manuscript so that they are identical.

Done.

9. We note that Figure 1 in your submission contain map images which may be copyrighted. All PLOS content is published under the Creative Commons Attribution License (CC BY 4.0), which means that the manuscript, images, and Supporting Information files will be freely available online, and any third party is permitted to access, download, copy, distribute, and use these materials in any way, even commercially, with proper attribution. For these reasons, we cannot publish previously copyrighted maps or satellite images created using proprietary data, such as Google software (Google Maps, Street View, and Earth). For more information, see our copyright guidelines: <http://journals.plos.org/plosone/s/licenses-and-copyright>.

We require you to either (1) present written permission from the copyright holder to publish these figures specifically under the CC BY 4.0 license, or (2) remove the figures from your submission:

We have created an updated version of Fig 1 that is now free from copyright restrictions. To accomplish this, we sourced the maps from the publicly available Natural Earth database (<https://www.naturalearthdata.com/downloads/10m-cultural-vectors/>). These maps were subsequently employed in QGIS v3.32, a free-open soft software (FOSS), for integrate the pie charts with specific geographic information. QGIS can be found in https://www.qgis.org/es/site/about/index.html. We modified the corresponding Methods section with this information:

“Geolocated pie chart maps were created using the Free and Open Source QGIS v.3.32, using the cartographic data sourced from Natural Earth Database (Quick start kit)."

Reviewer #1:

The author can detail on the sample collection - If all positive cultures from 17 states between 1998-2021 was analyzed in this study or it had any other preselection criteria.

The strain collection obtained includes more than 500 isolates. Samples reported in this study were pre-selected for relevant drug resistance phenotypes. We have included a comment on this regard in the methodos and results sections.

2.The authors have done the phenotypic dst for 1st line drugs alone to look into the pDST and gDST correlation with only few strains for looking into the correlation. This interpretation may change with more no.of strains being analysed.

We agree that the more samples we include in this analysis, the interpretation of the concordance analysis of pDST and gDST will change. As we pre-selected our sample dataset regarding the pDST results, the conclusion is conditionate. We have edited the manuscript to clarify this conclusion in the Discussion section:

“We acknowledge the limited sample size in our study and advocate for the inclusion of genomes from underrepresented geographic regions. We also recommend implementing technical controls to enhance the accuracy of antibiotic resistance detection and the use of random sampling methods. This will allow for a more confident assertion that Mexico has a higher discrepancy between genotyping and phenotyping DST compared to other regions.

3. The no of AwR mutations from the catalogue that were susceptible to the respective 1st line drugs in this study can be looked into in terms of these mutations that were categorized as AwR based on Expert rules in the WHO Catalogue and discussed.

Thanks for this valuable observation. We added some additional information about this topic within the Discussion section, as well as we updated the series of reference below that section.

*“Additionally, our Mtb dataset includes samples that show disparities in both SR and RS types, as detailed in S6 Table. Within this set, several AwR mutations were identified in H- and R-susceptible strains. For example, two samples in our dataset displayed an H-susceptible phenotype but encoded one of the most prevalent and statistically significant variants associated with H-resistance: katG Ser315Thr [6]. This particular mutation has been frequently reported globally in H-resistant samples [55, 56]. However, it's important to note that a 2002 study initially identified three Mtb samples as phenotypically H-susceptible, despite encoding the katG Ser315Thr mutation [57]. When the DST experiment was repeated, these samples were reclassified as H-resistant. This highlights the intricacies involved in interpreting susceptibility profiles and emphasizes the importance of rigorous validation using larger sample sets.”*

4. Based on the findings from this study, the need for analysing country specific data set and analysis framework for the mutations association with drug resistance can be recommended to help with a meaningful recommendation at the global level.

Thanks for the reviewer comment. We have included a couple of sentences on this regard in the discussion section.

*“Despite the limited number of samples analyzed in our study, our study highlights the necessity to examine data specific to individual countries and to adopt an analysis framework for the correlation between mutations and drug resistance. Such studies are essential for offering impactful recommendations on an international scale. Furthermore, our study contributes to a better understanding of the sublineages circulating in Mexico and their potential for their inclusion in global databases and comprehensive studies of drug resistance.”*

Reviewer #2:

Congratulation Paulina M et al for the nice study in the field of drug resistant tuberculosis. The study on the genomic epidemiology of drug-resistant Mycobacterium tuberculosis distributed in Mexico is an important contribution to the field of tuberculosis research. The authors have undertaken a comprehensive analysis using genomic data to shed light on the prevalence, distribution, and potential transmission patterns of drug-resistant strains of M. tuberculosis in the Mexico. Authors have also performed whole- Genome Sequencing of 85 isolates of M. tuberculosis isolated from different parts of Mexico. The results of this study could have significant implications for tuberculosis control strategies and public health interventions in Mexico.

The methodology appears to be sound and well-validated, I am fully agree on this .

The findings of the research has the potential to contribute valuable information to global efforts in combating tuberculosis and drug resistance. Sharing insights on the genomic diversity and transmission patterns of M. tuberculosis strains in Mexico can aid in a better understanding of the global dissemination of drug resistance.

Although the study focused on genomic analysis, it would be beneficial to include experimental data to validate drug resistance mechanisms identified through genomics. This would strengthen the overall conclusions and interpretations of the study. Furthermore, geospatial data is lacking. Addition of this data provides regional variations in drug-resistant tuberculosis distribution and identify specific hotspots of DR TB, which will in fact guide targeted interventions.

Please include details clinical information (e.g., treatment outcomes, patient demographics) with genomic data can provide a more comprehensive view of the epidemiological dynamics of drug-resistant tuberculosis in Mexico. This would offer valuable insights into risk factors and patient-specific management strategies.

It is suggested for sharing the raw genomic data through public repositories.

The study on genomic epidemiology analysis of drug-resistant Mycobacterium tuberculosis distributed in Mexico represents a significant contribution to the field of tuberculosis research. The authors have conducted a thorough investigation, and their findings have important implications for public health. Addressing the suggestions for improvement would strengthen the study's overall impact and contribute to its broader relevance and applicability in combating drug-resistant tuberculosis both in Mexico and globally. Therefore, I would like to recommend this study for publication after reviewing the reviewer suggestion.

Thank you

Thanks for the kind reviewer comments. We would like to say that we have included experimental drug resistance data determined in the laboratory for each strain, which can be readily accessed in **S2 Table** (Supporting information), and the NCBI BioSample database under the format “Pathogen: clinical or host-associated; version 1.0”, with the BioProject accession number: **PRJNA824124**. To enhance accessibility, we have included a column in S1-3 Tables containing the SRA accession number for each sample.

Regarding geographical information, we have also modified by adding “latitude” and “longitude” columns in S1 Table, providing the coordinates for each sampling location. Unfortunately, detailed data pertaining to treatment outcomes and other patient demographics were unavailable for our sample dataset. However, we already had included information on pertinent risk factors, such as Type-2 diabetes and HIV comorbidities, which is specified in S1 Table (Supporting information).
